# Synaptic Transmission from Horizontal Cells to Cones Is Impaired by Loss of Connexin Hemichannels

**DOI:** 10.1371/journal.pbio.1001107

**Published:** 2011-07-19

**Authors:** Lauw J. Klaassen, Ziyi Sun, Marvin N. Steijaert, Petra Bolte, Iris Fahrenfort, Trijntje Sjoerdsma, Jan Klooster, Yvonne Claassen, Colleen R. Shields, Huub M. M. Ten Eikelder, Ulrike Janssen-Bienhold, Georg Zoidl, Douglas G. McMahon, Maarten Kamermans

**Affiliations:** 1 Research Unit Retinal Signal Processing, The Netherlands Institute for Neuroscience, Amsterdam, The Netherlands; 2 Department of Biological Sciences, Vanderbilt University, Nashville, Tennessee, United States of America; 3 Department of Biomedical Engineering, Eindhoven University of Technology, The Netherlands; 4 Department of Neurobiology, University of Oldenburg, Oldenburg, Germany; 5 Department of Neurobiology and Behavior, SUNY at Stony Brook, Stony Brook, New York, United States of America; 6 Department of Neuroanatomy and Molecular Brain Research, Ruhr University, Bochum, Germany; 7 Department of Cytology, Ruhr University, Bochum, Germany; 8 Department of Neurogenetics, Academic Medical Center, University of Amsterdam, Amsterdam, The Netherlands; University of Washington, United States of America

## Abstract

In the vertebrate retina, horizontal cells generate the inhibitory surround of bipolar cells, an essential step in contrast enhancement. For the last decades, the mechanism involved in this inhibitory synaptic pathway has been a major controversy in retinal research. One hypothesis suggests that connexin hemichannels mediate this negative feedback signal; another suggests that feedback is mediated by protons. Mutant zebrafish were generated that lack connexin 55.5 hemichannels in horizontal cells. Whole cell voltage clamp recordings were made from isolated horizontal cells and cones in flat mount retinas. Light-induced feedback from horizontal cells to cones was reduced in mutants. A reduction of feedback was also found when horizontal cells were pharmacologically hyperpolarized but was absent when they were pharmacologically depolarized. Hemichannel currents in isolated horizontal cells showed a similar behavior. The hyperpolarization-induced hemichannel current was strongly reduced in the mutants while the depolarization-induced hemichannel current was not. Intracellular recordings were made from horizontal cells. Consistent with impaired feedback in the mutant, spectral opponent responses in horizontal cells were diminished in these animals. A behavioral assay revealed a lower contrast-sensitivity, illustrating the role of the horizontal cell to cone feedback pathway in contrast enhancement. Model simulations showed that the observed modifications of feedback can be accounted for by an ephaptic mechanism. A model for feedback, in which the number of connexin hemichannels is reduced to about 40%, fully predicts the specific asymmetric modification of feedback. To our knowledge, this is the first successful genetic interference in the feedback pathway from horizontal cells to cones. It provides direct evidence for an unconventional role of connexin hemichannels in the inhibitory synapse between horizontal cells and cones. This is an important step in resolving a long-standing debate about the unusual form of (ephaptic) synaptic transmission between horizontal cells and cones in the vertebrate retina.

## Author Summary

Contrast enhancement is a fundamental feature of our visual system, initiated at the first synaptic connections in the retina. These are the synapses between photoreceptors (rods and cones) and their targets, horizontal cells and bipolar cells. Horizontal cells receive input from many cones and subsequently send a feedback signal to photoreceptors. Bipolar cells, however, receive direct input from only a few photoreceptors, but also receive indirect inhibitory input from surrounding cones via the horizontal cell feedback pathway. This organization induces the classic center/surround organization of bipolar cells and is considered the first step in contrast enhancement. Exactly how horizontal cells send feedback signals to photoreceptors has remained a mystery, however. One hypothesis posits that connexin hemichannels are involved. In this study, we tested this hypothesis using mutant zebrafish that lack connexin hemichannels specifically in horizontal cells. Our electrophysiology experiments showed that feedback is indeed reduced in these mutants, confirming that connexin hemichannels play an important role in feedback from horizontal cells to cones. In addition, we find that these mutant fish have decreased contrast sensitivity at a behavioral level, illustrating that functionally relevant contrast enhancement begins at the first synapse of the visual system.

## Introduction

In the vertebrate retina, photoreceptors project to horizontal cells and bipolar cells. Horizontal cells are mediating lateral inhibition in the outer retina, a process that is thought to be involved in contrast enhancement. Horizontal cells are electrically coupled by gap-junctions, thus integrating their input spatially. This integrated signal is fed back negatively to photoreceptors. The effect of this feedback signal is a modulation of the Ca^2+^-current (*I_Ca_*) of the cones [1] and rods [2]. Hyperpolarization of horizontal cells shifts *I_Ca_* to more negative potentials, thus leading to an increase of Ca^2+^-influx and subsequently an increase in glutamate release by the cones. Although there is general agreement about this pathway, the underlying mechanism is a matter of debate [3]–[6].

For zebrafish, feedback from horizontal cells to cones has been suggested to depend on Cx55.5 hemichannels located at the tips of the horizontal cell dendrites [3],[7]. Current flowing through these hemichannels and the intersynaptic space induces a local voltage drop near the voltage dependent Ca^2+^-channels of the cones. This voltage drop depends on the horizontal cell membrane potential (V_HC_), thus making *I_Ca_* and the glutamate release from the cones also dependent on V_HC_ [3],[8],[9]. This interaction, where modulation of the extracellular potential leads to cell-cell communication, is called an ephaptic interaction.

The lack of specific pharmacology for connexins creates challenges for testing this hypothesis. Therefore, we generated a zebrafish that lacks Cx55.5 hemichannels and studied the negative feedback pathway from horizontal cells to cones. We found that Cx55.5 is mediating a major part of the feedback signal from horizontal cells to cones and that the lack of Cx55.5 hemichannels leads to reduced contrast sensitivity. This study is therefore the first study actually demonstrating the involvement of horizontal cells in contrast sensitivity at the behavioral level.

## Results

### Morphological Analysis of Cx55.5 (C54X) Mutant Zebrafish

**Figure 1:**
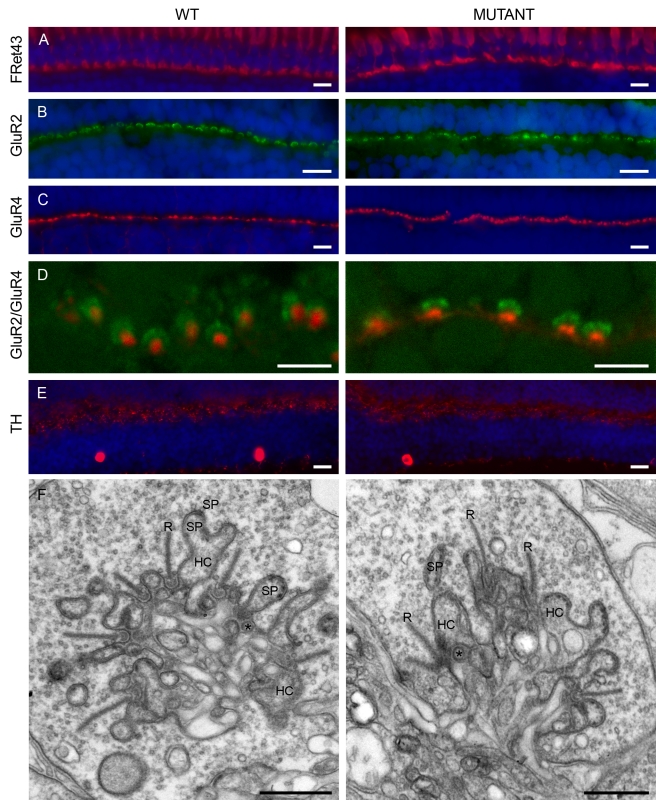
Localization of cellular markers in retinas from wild-type and mutant zebrafish. The organization of the outer retina was evaluated by the distribution of a number of cellular markers for double cones (A, FRet43) in red, horizontal cells (B, GluR2) in green, OFF-bipolar cells (C, GluR4) in red, and interplexiform cells (E, TH) in red. The blue stain is the nuclear marker DAPI. No difference in the distribution of these markers could be observed. In both preparations, GluR2 forms horseshoe-like structures in the OPL (B, D). Previous studies have shown that these structures are horizontal cell dendrites invaginating the cone terminal [57],[58]. GluR4 labeling is present as small puncta at the level of the OPL (C, D). These puncta were identified as the tips of the dendrites of OFF bipolar cells [57],[58]. (E) TH labels interplexiform cells and the synapses these cells make onto horizontal cells. The immunoreactivity pattern is similar in wild-type and mutant retinas. These results indicate that all major neuron types in the OPL have developed normally. (A to E): Scale bar = 10 µm. (F) Ultrastructure of the cone synaptic terminal in wild-type (left) and mutant (right) retinas. No differences were found in the ultrastructural organization of the cone synaptic terminal. In both animal models, the characteristic shape of the synaptic triad was easily identified. R, synaptic ribbon; Sp, spinules; HC, horizontal cell; * bipolar cell. Scale bar = 0.5 µm.

Connexins are proteins with four transmembrane domains, two highly conserved extracellular loops, one intracellular loop, and a long C-terminal end. To eliminate Cx55.5 function, we generated a zebrafish with a stop codon in the first extracellular loop of Cx55.5(C54X) using the TILLING technique (see Methods). The site of this mutation prevents the formation of a functional protein. The mutants develop normally. Body length (wild-type: 22±3 mm, *n* = 10; mutant: 22±1 mm, *n* = 10; *p*>0.05) and eye size (wild-type: 2.1±0.2 mm, *n* = 40; mutant: 2.1±0.2 mm, *n* = 41; *p*>0.05) did not differ significantly. Also the cell density in the outer nuclear layer (ONL) (*p* = 0.73), the inner nuclear layer (INL) (*p* = 0.80), and the ganglion cell layer (GCL) (*p* = 0.09) were equal. Finally, the localization of cell-specific markers was studied. The immunoreactivity of FRet43 (double cones), GluR2 (horizontal cell dendrites), GluR4 (OFF-bipolar cell dendrites), and TH (interplexiform cells) did not differ between wild-type and mutant and the ultrastructure of the cone synaptic terminal was normal (Figure 1).

**Figure 2:**
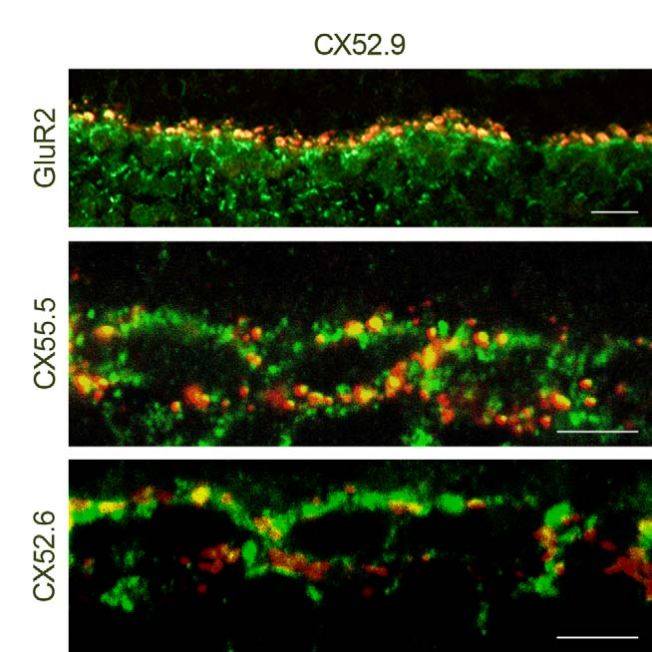
Cx52.9 co-localizes with GluR2, Cx55.5, and Cx52.6 in zebrafish retinas. The top panel shows co-localization of the Cx52.9 (green) antibody and GluR2-IR (red). GluR2 labels the tips of horizontal cell dendrites. This co-localization shows that Cx52.9 is present at the horizontal cell dendrites. Middle panel: Double labeling of Cx52.9 (green) and Cx55.5 (red). Red, green, and yellow plaques can be found, indicating that in some (parts) of the gap-junctions Cx55.5 co-localizes with Cx52.9. The bottom panel shows that there is co-localization of Cx52.6 (red) and Cx52.9 (green). Also for this combination, areas of co-expression of the two connexins can be found. This means that the three connexins co-localize in gap-junctions between horizontal cells. Scale bar = 10 µm (top panel). Scale bar = 5 µm (middle and bottom panel).

**Figure 3:**
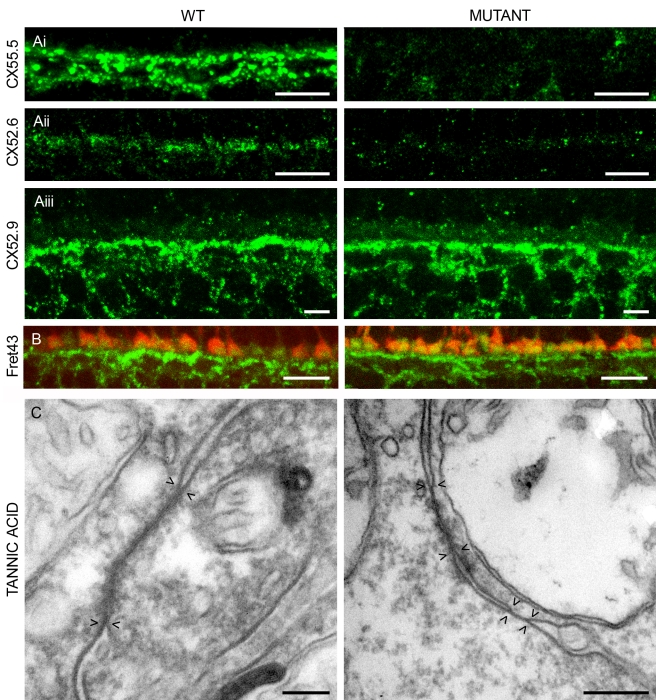
Localization and expression of connexins expressed by horizontal cells in wild-type and mutant zebrafish. (A) Immunocytochemical staining with antibodies against Cx55.5 (i), Cx52.6 (ii), and Cx52.9 (iii). Cx55.5-IR and Cx52.6-IR are absent in the mutant, whereas Cx52.9-IR remains present or even becomes stronger. Scale bar = 10 µm. (B) Double labeling of Cx52.9 (green) and FRet43 (red), a label for double cones. The Cx52.9-IR and FRet43-IR are closely associated, indicating that Cx52.9-IR is present in the dendrites of horizontal cells invaginating the cone synaptic terminal in both wild-type and mutant. Scale bar = 10 µm. (C) Tannic acid staining of gap-junctions in wild-type and mutant retinas. Pairs of arrow heads indicate the extent of the gap-junction. Often the gap-junctions in the mutant seem to be “split” as is illustrated in this figure. On average, gap-junctions are smaller in the mutant retinas. Scale bar = 0.5 µm.

**Figure 4:**
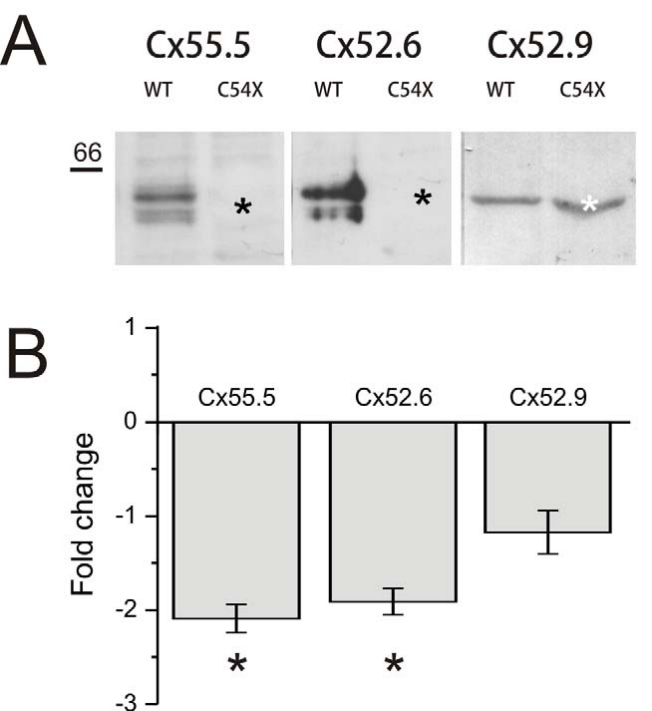
qPCR and western blot data confirm absence of Cx55.5 and Cx52.6. (A) Immunoreactivity patterns of Cx55.5, Cx52.6, and Cx52.9 antibodies in membrane samples of wild-type and C54X mutant zebrafish retinas. On Western blots derived from 8–10% SDS-PA gradient gels on which membrane samples (40 µg of each) of wild-type and mutant zebrafish retinas were separated, the Cx55.5 and Cx52.6 detected a double band with a molecular weight in the expected range (black asterisk) in the wild-type, but not the mutant retina (black asterisk). In contrast, the expression of Cx52.9 protein appeared to be unaffected in the mutant (white asterisk) and a protein with the appropriate molecular weight of Cx52.9 was detected in both membrane samples, wild-type and mutant. (B) qPCR experiments revealed a significant down regulation of Cx55.5 and Cx52.6 mRNA in mutant fish (*p*<0.001). Steady state Cx52.9 mRNA levels were not affected.

Immunoreactivity of at least three connexins (Cx52.6, Cx52.9, and Cx55.5) could be detected in the zebrafish retina (Figures 2 and 3A, left). These connexins were located in large plaques in the outer plexiform layer (OPL) and the upper part of the INL. Double labeling experiments confirmed that at least Cx52.9 co-localizes in gap-junctional plaques with both Cx55.5 and Cx52.6 (Figure 2). Co-localization of Cx55.5 and Cx52.6 was not studied due to the lack of appropriate antibodies. In the mutant (Figure 3A, right), both Cx55.5-IR and Cx52.6-IR were absent, indicating that these two connexins are co-regulated. Cx52.9-IR appeared normal. Western blot analysis confirmed that Cx55.5 and Cx52.6 were absent in the mutant retina (Figure 4A). Gap-junctions between horizontal cells were present in both wild-type and mutant (Figure 3C). Quantification of the gap-junctions showed a significant reduction in size (wild-type: 151±6.4 nm, *n* = 50; mutant: 104±4.8 nm, *n* = 50; *p*<0.001) (see Material and Methods) and a significant increase in number (wild-type: 0.061±0.010 gap-junction/µm^3^, *n* = 7; mutant: 0.094±0.011 gap-junction/µm^3^, *n* = 7; *p* = 0.046). These numbers suggest that the reduction in gap-junction size is compensated by an increase in gap-junction number. Based on these numbers, estimates of the total gap-junction surface in wild-type and mutant were made by assuming circular gap-junctional plaques. The total-gap-junctional surface did not differ significantly between wild-type and mutant (wild-type: 2,166±380 µm^2^/µm^3^; mutant: 1,590±221 µm^2^/µm^3^), suggesting that the total coupling conductance between horizontal cells was equal. Connexin expression on the horizontal cell dendrites differed between wild-type and mutant. In wild-type zebrafish, Cx55.5 and some Cx52.6 was expressed at the tips of the horizontal cell dendrites [3],[7], but this labeling was absent in the mutants. Cx52.9-IR on the horizontal cell dendrites in mutants remained present or might have increased slightly (Figure 3B). This shift in connexin expression was also observed at the RNA level. RNA levels of Cx55.5 (*p* = 0.035) and Cx52.6 (*p* = 0.02) were significantly down regulated in the mutant, whereas the RNA level of Cx52.9 (*p* = 0.49) was not (Figure 4B).

### Feedback Measurements in Horizontal Cells

**Figure 5:**
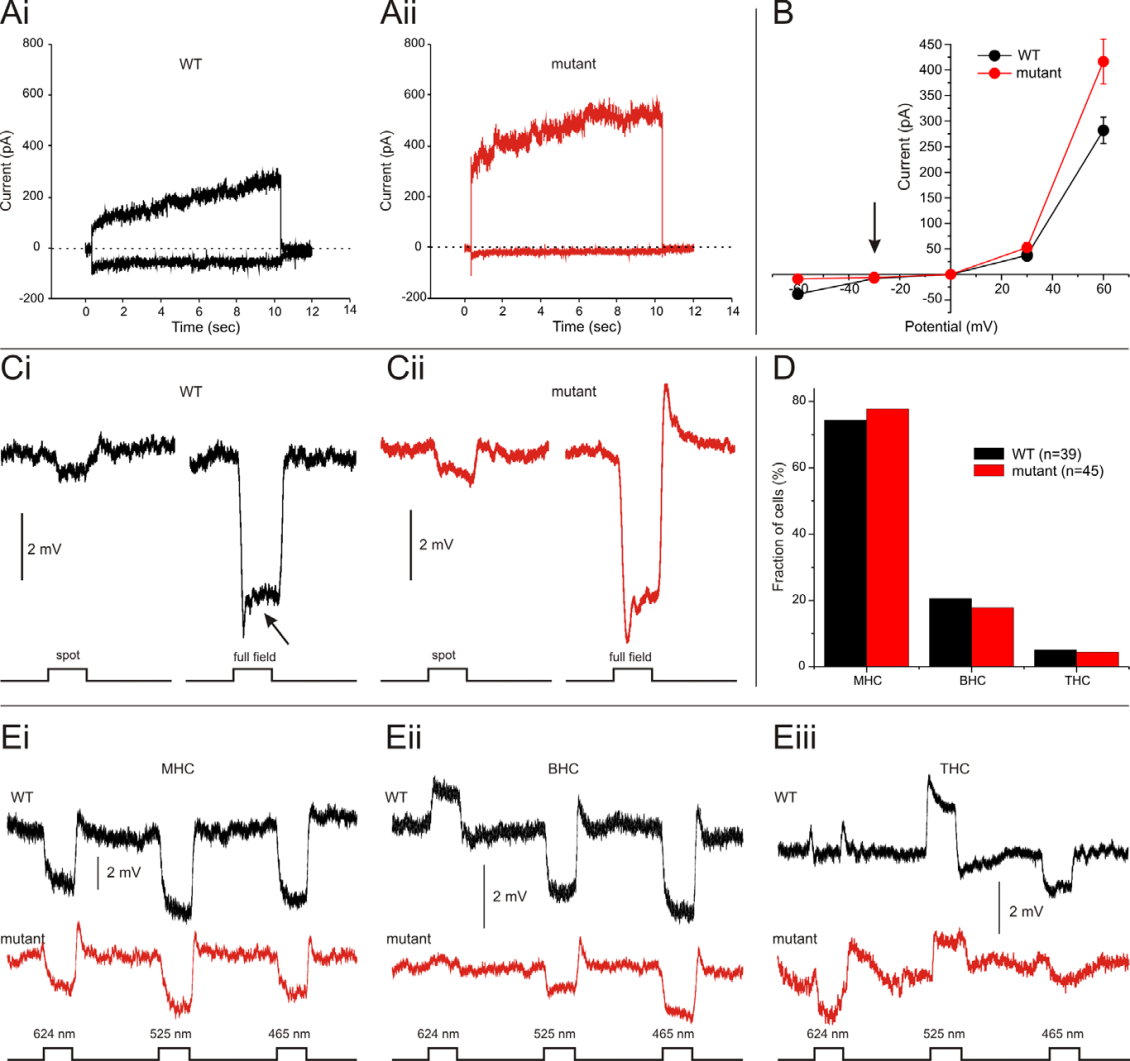
Horizontal cell properties in wild-type and mutant zebrafish. (A) *I_hemi_* in wild-type (black) and mutants (red) differ significantly. Whole cell currents of a dissociated horizontal cell when stepped to −60 mV and +60 mV, respectively. In the mutant, the inward *I_hemi_* at −60 mV is reduced, whereas the outward current at +60 mV is enhanced. (B) IV relation of *I_hemi_* in wild-type and mutants. *I_hemi_* is minimally affected around V_HC_ in the dark (−30 mV, arrow), is reduced at negative potentials, and is increased at positive potentials. (C) Responses to 500 ms flashes of 200 µm spot and full-field stimuli. The spot/full-field ratio did not differ between wild-type and mutant. (D) Distribution of horizontal cell classes in wild-type and in mutant fish did not differ significantly. (E) Three examples of spectrally coded horizontal cell responses in wild-type and mutants. The retina was stimulated with 500 ms full-field flashes of 624, 525, and 465 nm light.

Hemichannel currents (*I_hemi_*) in horizontal cells are affected in the mutant. *I_hemi_* was isolated and quantified in dissociated horizontal cells by whole-cell voltage clamp. The non-connexin steady-state membrane currents of isolated zebrafish horizontal cells are composed of inward and outwardly rectifying K^+^ currents that are blocked by internal Cs^+^ [10]. Here, horizontal cell K^+^ currents were blocked with internal Cs^+^ and tetraethylammonium (TEA) and hemichannel currents were evoked by positive or negative voltage steps from a holding potential of 0 mV in low Ca^2+^ medium to increase their amplitude [11],[12]. In horizontal cells of wild-type zebrafish, the currents recorded under these conditions exhibited the signature characteristics of hemichannels, non-selective outwardly rectifying currents that reversed at 0 mV [11],[12]. Wild-type cells showed a large outward current at positive potentials and a smaller inward current at negative potentials (Figure 5Ai, B). In horizontal cells of the mutants, the outward current at 60 mV was enhanced (wild-type: 282±26 pA, *n* = 11; mutant: 416±44 pA, *n* = 8; *p* = 0.012) while the inward current at −60 mV was reduced (wild-type: −38.8±6 pA, *n* = 11; mutant: −8.6±1.0 pA, *n* = 8; *p*<0.001) (Figure 5Aii, B). No significant change in the hemichannel current was found at −30 mV, which is close to V_HC_ in the dark (wild-type: −8.0±1.0 pA, *n* = 5; mutant: −6.4±1.0 pA, *n* = 5; *p* = 0.57) (Figure 5B, arrow). 100 µM cobalt is very effective in blocking negative feedback from horizontal cells to cones [13]–[16]. Next we tested whether the hemichannel current in horizontal cells can be blocked in this way. Application of 100 µM cobalt produced a reduction in the inward current in wild-type horizontal cells similar in magnitude to the reduction in inward current observed in Cx55.5 mutant horizontal cells (Figure S1). The voltage dependence of the hemichannel currents in dissociated horizontal cells of wild-type and mutant zebrafish are in agreement with the known voltage-dependence of Cx55.5 and Cx52.6 hemichannels [7],[17].

Is horizontal cell function affected in the mutant? Horizontal cells in the isolated retina responded with a hyperpolarizing response to 500 ms full-field blue light stimuli. The V_HC_ in the dark (wild-type: −30.4±2.2 mV, *n* = 35; mutant: −28.5±1.9 mV, *n* = 30; *p* = 0.53) and the response amplitude (wild-type: −2.2±0.4 mV, *n* = 35; mutant: −1.5±0.3 mV, *n* = 30; *p* = 0.21) did not differ between wild-type and mutants. Receptive field sizes of horizontal cells were estimated by stimulating the retina with a 200 µm spot and a full-field stimulus (Figure 5C). The spot/full-field ratio in the wild-type (0.34±0.09, *n* = 15) and mutants (0.33±0.10, *n* = 11) were equal (*p* = 0.91), indicating that the receptive field size had not changed. The overshoot response at light offset seemed to be larger in the mutant, but was not analyzed any further in the present paper.

Next it was determined whether negative feedback from horizontal cells to cones was affected in the mutant. Overall, the roll-back responses of horizontal cells (Figure 5Ci, arrow), an indirect measure of feedback [18]–[21], were highly variable and small in both wild-type and mutants. Many cells, even in wild-type, did not show any sign of a rollback response (see also Connaughton and Nelson [22]). This is reflected in the average size of the rollback response, which did not differ significantly from zero for both the wild-type and the mutant (wild-type: 1.4±6.8%, *n* = 35; mutant: 6.2±5.9%, *n* = 30; *p* = 0.30; *p* = 0.78). Furthermore, they did not differ from each other (*p* = 0.60). Another measure of feedback is the spectral sensitivity of horizontal cells [20],[22]–[24]. Horizontal cells can be classified spectrally as monophasic horizontal cells (MHC) that hyperpolarize to all visible wavelengths, biphasic horizontal cells (BHC) that hyperpolarize to short and depolarize to long wavelength light, and triphasic horizontal cells (THC) that hyperpolarize to short and long wavelength light but depolarize to middle wavelength light [22],[25]–[28]. Tetraphasic horizontal cells have been reported [22], but are not included in this study. Figure 5D shows the distribution of horizontal cell types, and Figure 5E shows horizontal cell responses to red, green, and blue full-field light stimuli, respectively. The distribution of horizontal cells in the mutant did not differ from wild-type (*p*>0.05), indicating that the wiring of the horizontal cell types developed normally in the mutants. The depolarizing light responses are purely feedback-driven responses, while the hyperpolarizing responses to blue light are due to the direct light responses of the cones [20],[22]–[24]. In zebrafish, some BHCs depolarize while others hyperpolarize in response to green light stimulation (Figure S2 and see Connaughton and Nelson [22]). All the depolarizing responses in the BHCs were strongly reduced in the mutant. To quantify this, we used the ratio between the depolarizing responses to red light and the hyperpolarizing responses to blue light as an estimate of feedback. As shown above, the average response amplitude to blue light stimuli did not differ between wild-type and mutant. However, the ratio between red and blue light responses in the BHCs was significantly smaller in the mutant (wild-type: −0.38±0.05, *n* = 8; mutant: −0.08±0.03, *n* = 5; *p* = 0.001; Figures 5Eii and S2). In the MHCs no difference in the ratio between red and blue light responses was found (wild-type: 0.67±0.11, *n* = 25; mutant: 0.87±0.11, *n* = 24; *p* = 0.20). This shows that the depolarizing response of the BHCs to red light was reduced relative to the response to blue light, indicating that feedback in the mutant was reduced. The depolarizing responses of the THCs seemed to be smaller as well, but statistical analysis could not be performed due to the low number of THCs encountered.

### Feedback Measurements in Cones

**Figure 6:**
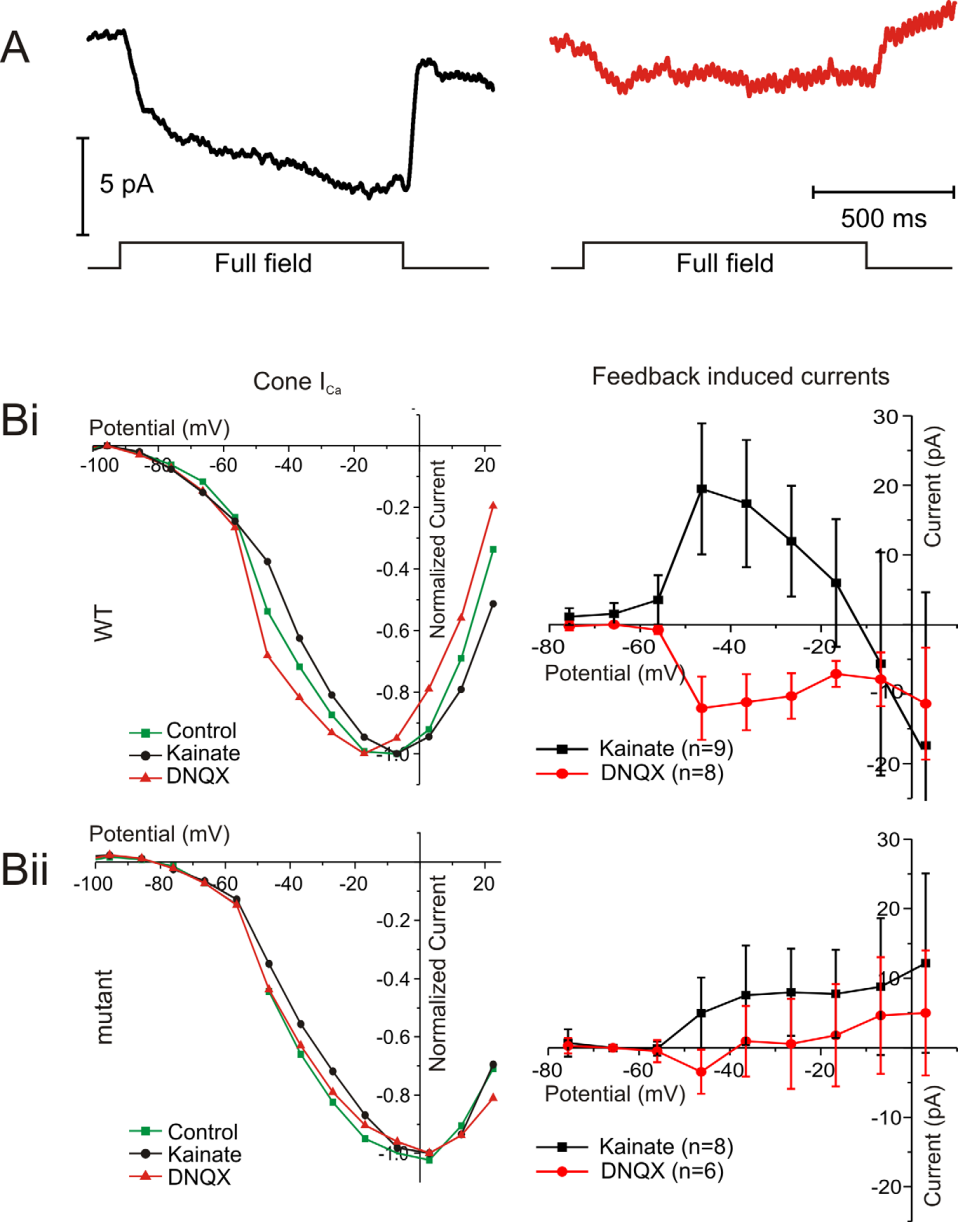
Feedback measured in cones is reduced in the mutant zebrafish. (A) Light-induced feedback responses in wild-type (left) and mutant (right) zebrafish cones. Horizontal cells were hyperpolarized by full-field 1 s flashes of light while the recorded cone was saturated with a 20 µm spot of bright light. (B) IV relation of a cone *I_Ca_* in control (green), 50 µM DNQX (red), and 30 µM KA (black) in wild-type (Bi, left) and mutant (Bii, left). Averaged DNQX- (red) and KA- (black) induced currents in wild-type (Bi, right) and mutants (Bii, right). The DNQX-induced current is significantly reduced in the mutant compared to the current in wild-type.

Next, we studied feedback responses in cones. Light-induced feedback responses were measured first. Cones were saturated with a 20 µm spot of light and 500 ms full-field stimuli were applied in addition. Such a protocol induces an inward current which is due to negative feedback from horizontal cells to cones [1],[3]. Figure 6A shows feedback-induced responses of wild-type (black) and mutant cones (red). On average the feedback-induced inward current in mutants was smaller than in wild-type cones (wild-type: −3.37±0.72 pA, *n* = 9; mutant 0.41±0.81 pA, *n* = 6; *p* = 0.004). The mutant feedback-induced currents did not differ significantly from zero (*p* = 0.635) while they did in the wild-type (*p* = 0.002).

To obtain a more reliable measure of feedback, the membrane potential of horizontal cells was manipulated without directly affecting cones by applying 50 µM 6,7-Dinitroquinoxaline-2,3(1H,4H)-dione (DNQX), a glutamate antagonist, or 30 µM Kainate (KA), a glutamate agonist [13],[29],[30]. Figure 6Bi (left) shows the cone *I_Ca_* in the control condition (green), in DNQX (red), and in KA (black). In wild-type, DNQX and KA shifted *I_Ca_* to more negative and more positive potentials, respectively. As in wild-type, KA shifted *I_Ca_* to more positive potentials in the mutant (Figure 6Bii, left). However, DNQX failed to shift *I_Ca_* of mutant cones to more negative potentials. To quantify the amount of feedback, the whole cell IV relation in control conditions was subtracted from the whole cell IV relation in DNQX or KA (Figure 6B, right) and the surface under the resulting curves was determined. Feedback induced by DNQX was significantly reduced in the mutant (wild-type: −369±117 pA*mV, *n* = 8; mutant: 121±72 pA*mV, *n* = 6; *p* = 0.007), while feedback induced by KA was not significantly reduced (wild-type: 527±281 pA*mV, *n* = 9; mutant: 240±197 pA*mV, *n* = 8; *p* = 0.43).

**Figure 7:**
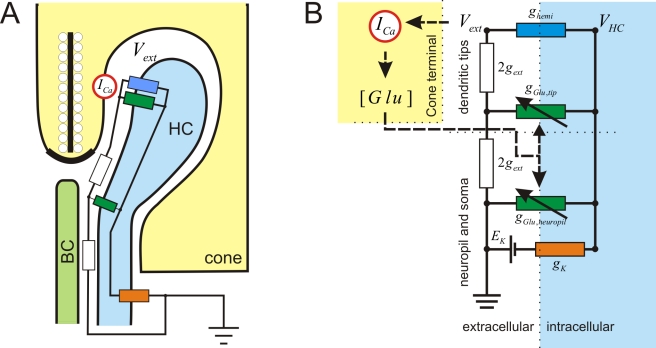
Model for ephaptic feedback. (A) Schematic drawing of the cone synaptic terminal. HC, horizontal cell; BC, bipolar cell; *I_Ca_*, cone Ca^2+^-current; *V_ext_*, potential in the synaptic cleft. (B) The feedback model formulated by Fahrenfort et al. [6] was used to evaluate the effect of changes in voltage dependence of horizontal cell hemichannels. Here we will give a short description of the main features, for a full description see Fahrenfort et al. [6]. The model consists of a simple resistive network. The model was used to evaluate the relation between the horizontal cell membrane potential (*V_HC_*) and the change in extracellular potential (*V_ext_*) with different numbers of hemichannels in the horizontal cell membrane. *V_ext_* is the potential in the synaptic cleft, *V_HC_* is the horizontal cell membrane potential, *g_hemi_* is the hemichannel conductance, *g_Glu, tip_* is the glutamate conductance at the tips of the horizontal cell dendrites, *g_Glu,__neuropil_* is the glutamate conductance of the horizontal cell dendrites in the neuropil, *g_K_*, is the potassium conductance of horizontal cells and *E_K_* is the equilibrium potential for potassium. [*Glu*] is the Glutamate-concentration in the synaptic cleft. *I_Ca_* is the cone Ca^2+^-current; g_ext_ is the conductance of the extracellular space in the synaptic complex.

**Figure 8:**
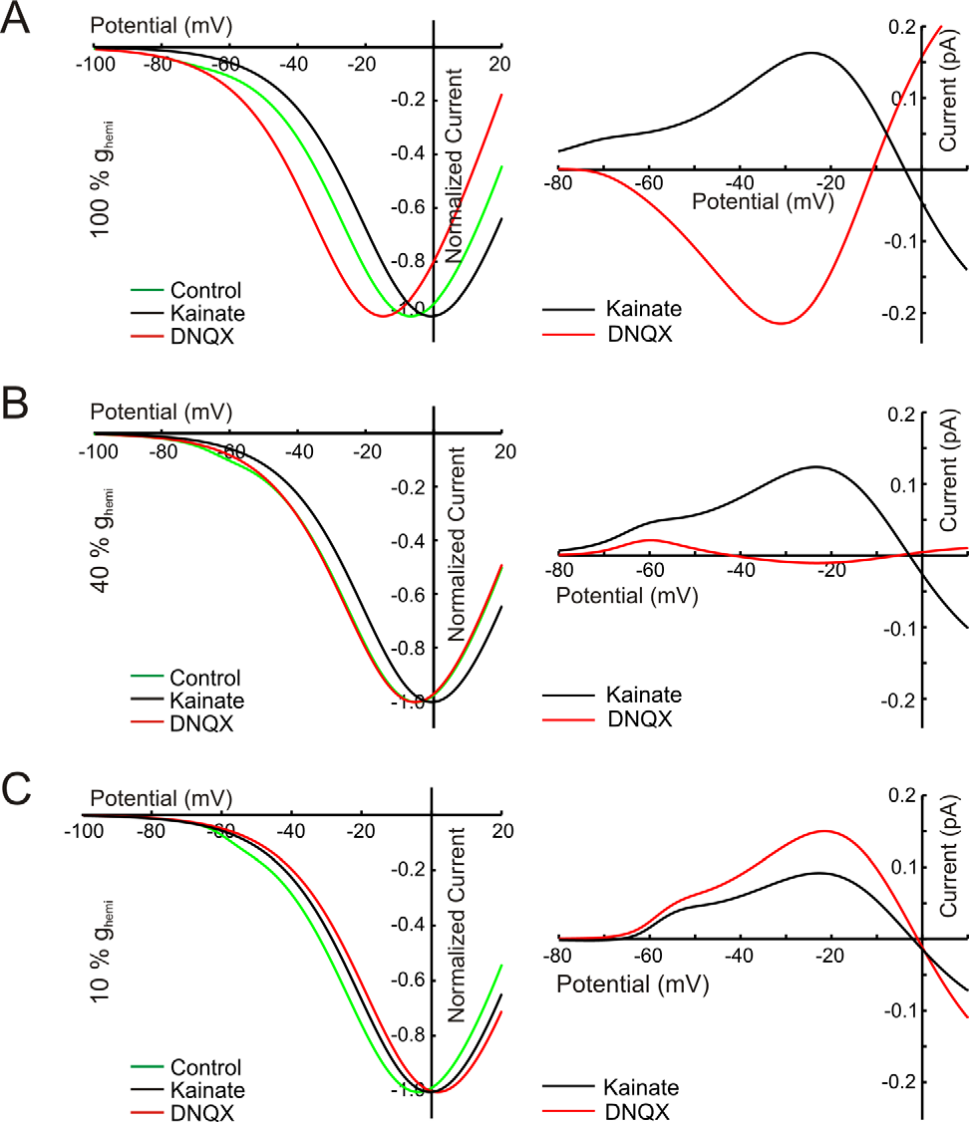
Model simulations of feedback induced shift of Ca-current in wild-type and mutant zebrafish. Left: The simulated IV relations of 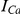 in control condition, DNQX, and KA, produced by the ephaptic feedback model [6]. Right: the DNQX- and KA-induced feedback currents. The value of 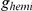 was varied from 100% (A) to 10% (C) of the wild-type value [6]. With reducing 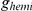, the DNQX-induced feedback currents decrease and finally reverse. The KA-induced feedback currents only decrease with decreasing 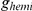.

To test whether a reduced *I_hemi_* could account for the decreased feedback in the mutants, we studied feedback-induced currents for different values of the hemichannel conductance (*g_hemi_*) in our quantitative feedback model (Figure 7) [6]. The DNQX-induced feedback currents were strongly reduced and eventually reverse with reduction of *I_hemi_* (Figure 8). In contrast, the KA-induced feedback remained almost intact. Both the experimental data and the model show that feedback is affected in a highly asymmetrical manner. At about 40% *g_hemi_*, the model mimics the experimental findings (Figure 6Cii), suggesting the presence of some residual *g_hemi_* in the mutants.

### Optokinetic Response of Larval Zebrafish

**Figure 9:**
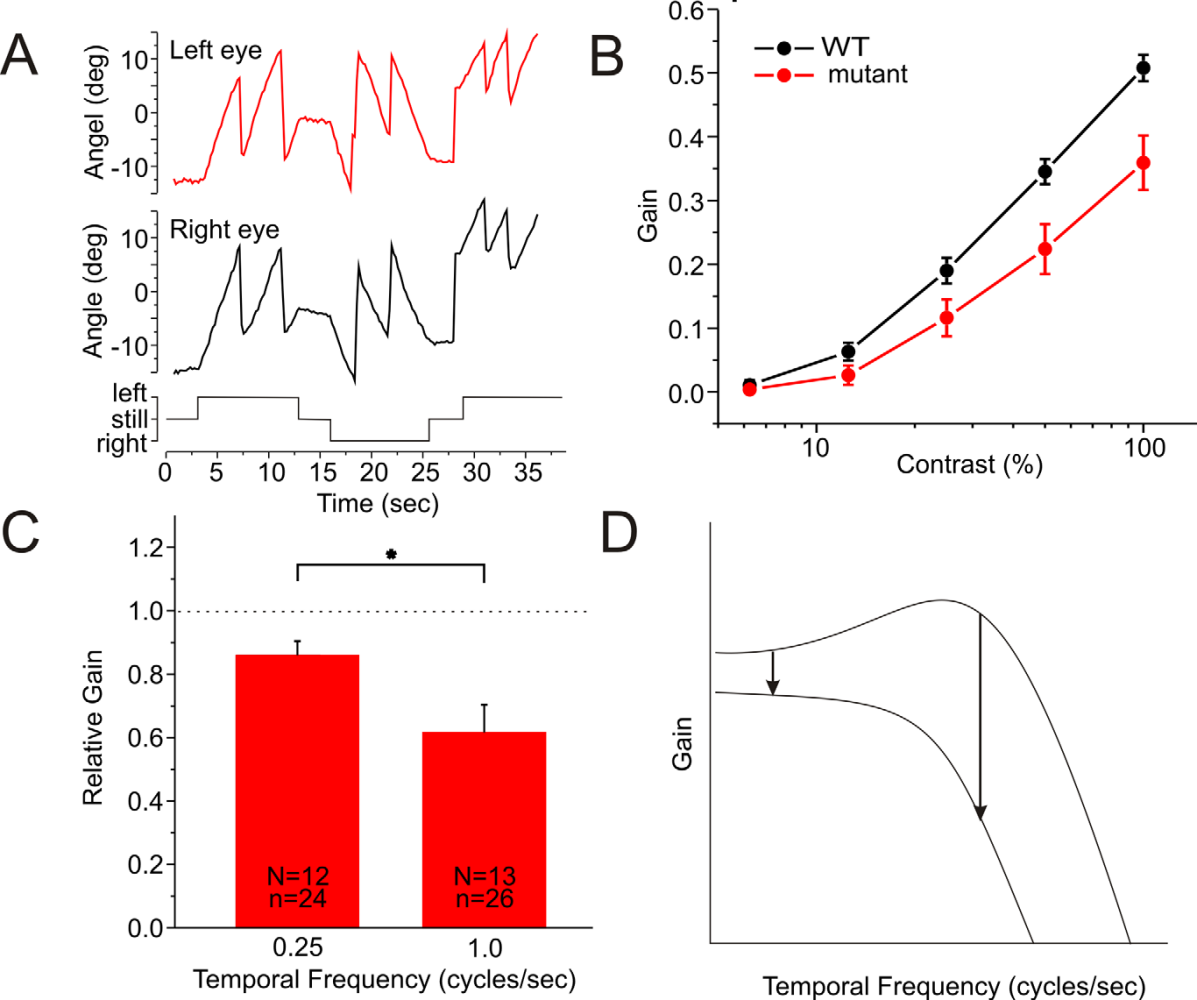
Optokinetic response of mutant zebrafish is reduced. (A) Eye movements of a wild-type zebrafish larva (black, right eye; red, left eye). Timing of the stimulus is indicated in the bottom trace. (B) Optokinetic gain as function of contrast for 13 wild-type (black) and 13 mutant (red) zebrafish. Over the whole contrast range the wild-type performed significantly better than the mutant. (C) In the mutant, reduction in optokinetic gain is stronger for high temporal frequencies (1.0 cycle per second) than for low temporal frequencies (0.25 cycle per second) (*p* = 0.017). (D) In wild-type the temporal frequency transfer function of cones to bipolar cells is a band-pass filter. The low frequency cutoff is due to negative feedback from horizontal cells. When removing this pathway and inducing an overall gain reduction, the transfer function changes into a low-pass filter. At low frequencies this transformation leads to a smaller loss in gain than for high temporal frequencies.

How would vision be affected by this mutation? We have shown previously that feedback has two components: subtractive and multiplicative [31]. The subtractive component subtracts the global mean activity of the cone output and the multiplicative component amplifies the remaining signal, scaling it such that it fits the bandwidth of the bipolar cells. The result is that the information about objects deviating from the surround is transmitted with high fidelity to the brain. Such a mechanism also generates a form of color-constancy [32],[33]. Without feedback the amount of information transmitted from the cones to the second order neurons would be reduced, thus lowering their contrast sensitivity and color-constancy. To test the first prediction, we measured the optokinetic response (OKR) of 5-d-old zebrafish larvae as function of contrast. OKR responses were generated with an LED-based optokinetic stimulator (Figure S3). This stimulator allows the projection of (sine-wave) patterns of light of various contrasts and colors and rotates these patterns with various velocities. Eye movements are recorded (Figure 9A) and the optokinetic gain is determined by dividing the eye movement velocity by the velocity of the stimulus. Figure 9B shows that for all contrasts the optokinetic gain was significantly reduced in mutant zebrafish compared to wild-type (*p*<0.01). Since Cx55.5 is exclusively expressed in retinal horizontal cells [7], these experiments show that negative feedback from horizontal cells to cones is important for contrast sensitivity. Finally, in a separate set of experiments, the temporal frequency dependence of the optokinetic gain was determined (Figure 9C). The gain reduction found in the mutants was stronger for high than for low temporal frequencies (*p* = 0.017). This is consistent with the notion that a negative feedback pathway enhances the synaptic gain [31] and acts as a band-pass filter. Removing negative feedback will reduce the gain and in addition convert the filter into a low pass filter (Figure 9D). The result is that high frequencies will be affected more by the gain reduction than low temporal frequencies.

## Discussion

The synaptic mechanism via which retinal horizontal cells feed back to photoreceptors has been a matter of intense debate. In the present study, we show that feedback is impaired in mutant zebrafish lacking Cx55.5, a connexin which is expressed specifically in horizontal cells. Reduction of feedback was observed in both horizontal cells and cones. Furthermore, we show now on a behavioral level that feedback from horizontal cells to cones plays a prominent role in contrast enhancement. Taken together, our observations show that connexin hemichannels play an important role in synaptic communication between horizontal cells and cones.

### Spectral Opponent Coding of Horizontal Cells

In this article, we show that feedback from horizontal cells to cones generates the spectral coding of horizontal cells; reduced feedback in the mutant zebrafish leads to a decreased depolarizing response to red light in BHCs. Fuortes and co-workers [34] and Stell and co-workers [23],[24] were the first to propose that the depolarizing responses in BHCs were due to negative feedback from horizontal cells to cones. More than 35 years later, we are now able to confirm this assumption by genetic means. The exact wiring between horizontal cells and cones still remains an issue of debate [20],[22]–[24],[35]. However, with genetically modified animals, such as the one presented here, together with animals that express GFP in specific types of horizontal cells, this issue may be resolved in the near future.

### Feedback Is Reduced But Not Absent in Mutant Zebrafish

In the mutant zebrafish lacking Cx55.5, feedback from horizontal cells to cones is significantly smaller, but not completely abolished. Our model simulations suggest that about 40% of the hemichannel conductance remains in the mutants. These hemichannels could be formed by other connexins such as Cx52.9, an ortholog of Cx55.5, or by Pannexin 1 (Panx1). Interestingly, Panx1 RNA was significantly up-regulated (2.33±0.6, *p* = 0.01) in the mutant, and Panx1-IR found on the horizontal cell dendrites [36] was also present in the mutant zebrafish (unpublished data). It is an intriguing possibility that the expression of another hemichannel forming protein, Panx1, might compensate for the lack of Cx55.5 hemichannels.

In the mutant zebrafish, we observed a significant reduction in the shift of *I_Ca_* in cones when horizontal cells were pharmacologically hyperpolarized, but only a small non-significant reduction when horizontal cells were depolarized. The model simulations show that a reduction in *g_hemi_* in itself can account for this asymmetric reduction of feedback. How does this work? In wild-type horizontal cells, hyperpolarization leads to an increase of *I_hemi_*. It has been proposed that this increased *I_hemi_* induces the shift of *I_Ca_* via an ephaptic interaction [3]. In the mutant, the hemichannel conductance at negative potentials is strongly reduced (Figure 5B). The result is that with hyperpolarization of the horizontal cells, *I_hemi_* does not increase but remains equal or even decreases. Due to this change, the ephaptic interaction will be absent or even reversed. Depolarization of horizontal cells leads to a reduction of the hemichannel current both in the wild-type and the mutant fish. Therefore, feedback due to horizontal cell depolarization might decrease slightly, but will never reverse in the mutant.

### Feedback and Horizontal Cell Coupling

In an ephaptic feedback model, the current that enters the horizontal cell via the hemichannels at the tips of the horizontal cell dendrites has to leave the horizontal cell at another place. In principle there are two routes for this: The current leaves the horizontal cell via the potassium channels [6] or the current leaves the horizontal cell via the gap-junctions to neighboring horizontal cells and leaves those neighboring horizontal cells via glutamate gated channels, hemichannels, and potassium channels. These two routes differ considerably. The main difference is that for homogenous stimuli, all horizontal cells will be polarized equally and thus prevent current flow through the gap-junctions. With hyperpolarization of the horizontal cells, the inward rectifying potassium current will activate and allows more current to leave the horizontal cells. The consequence is that for such stimuli the properties of the potassium channels also affect the properties of feedback. For small spots or edges, on the other hand, the second route might be the preferred route. For small spots horizontal cells do not hyperpolarize strongly, limiting the amount of current that can leave the horizontal cell via the potassium channels. Now a major part of the current will flow via the gap-junctions to neighboring horizontal cells. The properties of feedback are for a large part dependent on the properties of the gap-junctions. To prevent interference of this pathway in our experiments, we have used full-field stimuli or pharmacological polarization of horizontal cells to induce feedback responses.

### Alternative Models for Feedback from Horizontal Cells to Cones

It has been shown that negative feedback from horizontal cells to cones is pH-dependent [4],[5]. At first sight, these results seem to be contradicting the ephaptic feedback hypothesis. However, as we have shown previously, the pharmacological manipulations used in these studies lead to intracellular pH changes as well [6]. Since connexin-hemichannels close with intracellular acidification, the findings supporting a pH-dependent mechanism are not in conflict with, but even support, the ephaptic feedback hypothesis [6],[37]–[40]. Nevertheless, although our data are well described by an ephaptic feedback mechanism, we cannot fully exclude that another process, independent of hemichannels [4],[5], mediates the remaining feedback. Our data predict that such an alternative mechanism will be most prominently active at potentials more depolarized than V_HC_ in the dark and can be blocked by gap-junction blockers [3].

In this article we show that in mutant zebrafish the roll-back responses of horizontal cells are not affected, whereas feedback is affected. In previous studies where the rollback response was used as measure for feedback, the size of the rollback response was compared within one cell in different pharmacological conditions [3],[41]. In this study, the mean of the rollback responses in different groups were compared. Due to the large variability in the size of the rollback response, the average rollback response was not significantly different from zero in both the wild-type and the mutant zebrafish. This indicates that the rollback response is not a very sensitive measure for feedback when comparing group means. Mouse horizontal cells express Cx57 [41]–[44]. Because the rollback response of horizontal cells did not differ in the Cx57^−/−^ knock-out mouse, it was concluded that Cx57 is not involved in feedback from horizontal cells to cones. Our experiments show that more rigorous testing of the feedback pathway is needed before reaching such a conclusion, leaving the possibility that feedback is affected in the Cx57^−/−^ mouse.

To our knowledge, this study is the first non-pharmacological intervention specifically affecting the horizontal cell to cone feedback system and shows that Cx55.5 hemichannels are essential in this pathway. It highlights the unconventional nature of this synapse, demonstrating that feedback can be blocked in a highly asymmetric manner. An ephaptic model fully predicts this specific aspect of feedback. This study adds to the evidence for an ephaptic feedback mechanism in which connexin hemichannels and possibly pannexin channels have a physiological function.

## Materials and Methods

All procedures conformed to the Association for Research in Vision and Ophthalmology statement for the use of animals in ophthalmic and vision research. All protocols were approved by the Stony Brook IACUC Committee on Animal Use in Research or were carried out under the responsibility of the experimental animal ethical committee of the Royal Netherlands Academy of Arts and Sciences (KNAW) (protocol NIN2006.40) in accordance with the Council of the European Communities Directive of 24 November 1986 (86/609/EEC) or in accordance with the National Institute of Health guideline for animal use.

### Animal Care

Wild-type zebrafish, *Danio rerio*, (TL and AB strains) were obtained from the Zebrafish International Resource Center (Eugene, OR; NIH-NCRR grant #RR12546). Male and female fish were maintained in aquaria at 28° to 28.5°C under a 14/10 h light/dark cycle. Zebrafish were fed two to four times a day on a diet of live arthemia (INVE Technologies, Dendermond, Belgium; INVE Aquaculture, Salt Lake City, UT) and dry food [Nippai Feed (Catvis, Netherlands); or a flake food mix of Aquatox flake food (Aquatic Ecosystems, Apopka, FL), Cyclopeeze (JEHMCO, www.jehmco.com), Hikari micropellets (Aquatic Ecosystems, Apopka, FL), and 300–500 micron Golden Pearl fish food (Brine Shrimp Direct, Ogden, UT) mixed in a ratio of 14∶1∶1.4∶1.4, respectively]. Zebrafish lines were propagated by natural mating initiated by the onset of light. Embryos were grown at 28° to 28.5°C in media containing 1×10^−5^% methylene blue (Sigma, St. Louis, MO) under standard conditions.

### Connexin 55.5 Mutant Fish

Mutant zebrafish were generated by target-selected inactivation (TILLING) at the Hubrecht Laboratory (Utrecht, The Netherlands) as a part of a European project (European Commission LSHG-CT-2003-503496) [45],[46]. In short: A library of ENU-mutagenized F1 zebrafish was generated and kept as a living stock. The DNA of these animals was screened for mutations in the Cx55.5 gene by use of CEL-I-mediated heteroduplex cleavage and subsequent resequencing. This process created a line of zebrafish with a cytosine to adenine mutation at nucleotide position 162 of the Cx55.5 coding sequence. This nucleotide substitution results in a premature stop codon at amino acid position 54, where a cysteine residue is present in wild-type zebrafish. Based on the predicted membrane topology, only one of the four transmembrane domains should be translated in the mutant. Zebrafish homozygous for this mutation, therefore, can be considered as Cx55.5 knock-outs. In order to remove unwanted ENU-induced mutations, the Cx55.5 mutant line was out-crossed to wild-type for at least four generations in order to remove unwanted ENU-induced mutations. Only homozygous mutants and closely related wild-type zebrafish were analyzed in this study.

### Immunohistochemistry

#### Primary antibodies

The anti-Cx52.9 antibody was generated against a 17 amino acid peptide (LRDHCPALRDGPADSSA) in the carboxy terminus of Cx52.9. Note that the carboxy termini are regions of high diversity among connexins. Antigenic peptide and affinity-purified chicken anti-Cx52.9 were prepared by Davids Biotechnology (Reynersburg, Germany). Antibody specificity was confirmed by Western blot and immunohistochemistry using preimmune sera with antigenic peptide as controls. The Cx52.6 and Cx55.5 antibodies were synthesized and validated as described in Shields et al. [7]. Mouse monoclonal antibodies against tyrosine hydroxylase (TH) and the ionotropic glutamate receptor GluR2 and GluR4 were obtained from Chemicon (Temecula, CA), and FRet43, reported to label double cones in zebrafish [47], was a kind gift from Ms. R. BreMiller (University of Oregon, Eugene, Oregon).

### Western Blots of Connexins Expressed by Horizontal Cells

Zebrafish retinas (28 wild-type and 24 mutant retinas including residual choroid and vitreous) were dissected in oxygenated Leibowitz's L-15 medium, pH 7.4, and homogenized in ice-cold homogenization buffer (50 mM Tris, 2 mM EGTA, 2 mM EDTA, 1 mM DTE, 1 mM PMSF), pH 7.4, containing protease and phosphatase inhibitor cocktails (Roche Diagnostics, Mannheim, Germany); membrane fractions were prepared and aliquots (40 µg of protein) of wild-type and mutant samples were dissolved in gel-loading buffer [48]. Proteins were separated by SDS-PAGE on 8%–10% gradient gels and transferred to nitrocellulose (Optitran BA-S85, Schleicher Schuell, Dassel, Germany). Incubations with primary antibodies (rabbit anti-zfCx55.5 diluted 1∶2,000, rabbit anti-zfCx52.6, and chicken anti-zfCx52.9 each diluted 1∶500) were carried out in TBS-Tween buffer (20 mM Tris/HCl, pH 7.4, 150 mM NaCl, 0.2% Tween-20) at 4°C overnight. After several washes in TBS-Tween, immunoreactive proteins were either visualized with horseradish peroxidase-conjugated goat anti-rabbit IgG (BioRad Laboratories, Munich, Germany; diluted 1∶3,000 in TBS-Tween with 2% powdered milk) and enhanced chemiluminescence detection (Pierce, Rockford, IL), or with alkaline phosphatase-conjugated rabbit anti-chicken IgG (Jackson ImmunoResearch Laboratories, West Grove, PA; diluted 1∶1,000 in TBS (50 mM Tris/HCl, 100 mM NaCl, 1 mM MgCl_2_), pH 7.4 with 2% powdered milk) and the NBT/BCIP/PMS developer system (0.03% nitrotetrazolium blue, 0.015% 5-bromo-4-chloro-3-indolyl phosphate p-toluidine, 0.0015% N-methyldibenzopyrazine methylsulfate in 100 mM Tris/HCl buffer, pH 9.5, containing 100 mM NaCl and 5 mM MgCl_2_).

### Light Microscopy

For Figures 1 and 2 the following procedure was used. Whole eyes or zebrafish larvae were fixed for 10 min at room temperature in 4% paraformaldehyde in 0.1 M phosphate buffer (PB, pH 6.5) followed by 10-min fixation in 4% paraformaldehyde in 0.1 M sodium carbonate buffer (pH 10.4). The tissue was rinsed (4 times, 10 min each) in PB (0.1 M, pH 7.4). The tissue was then cryoprotected at room temperature in PB containing 12.5% sucrose for 45 min and then in PB containing 25% sucrose for at least 1 h. The tissue was embedded in Tissue Tek (Sakura Finetek Europe, Zoeterwoude, The Netherlands) in an aluminum foil boat and frozen on dry ice or in liquid nitrogen. 10 µm-thick cryosections were mounted on poly-L lysine-coated glass slides, dried, and stored in a standard freezer at −20°C. Sections were washed in PBS (10 mM Na_2_HPO_4_, 2.5 mM NaH_2_PO_4_, 120 mM NaCl, 2.7 mM KCl) for 10 min (two times) and blocked in 2% normal goat serum (NGS; Jackson Immunoresearch, West Grove, PA) in PBS for at least 20 min. Retinal sections or whole retinas were incubated with primary antibody first for 2 h at room temperature and then for 24–48 h at 4°C in PBS containing 0.3% Triton X-100, 0.02% sodium azide, and 5% NGS. Primary antibodies were diluted as follows: FRet43: 1∶200; tyrosine hydroxylase: 1∶400; GluR2: 1∶75; GluR4: 1∶10; Cx52.6: 1∶1,000; Cx55.5: 1∶10,000 or 1∶20,000; Cx52.9 1∶100. After three rinses (15 min each) in PBS, sections were either incubated in goat anti-mouse Alexa488- or Alexa568- (Invitrogen, Karlsruhe, Germany) and goat anti-rabbit Cy3- (Jackson Immunoresearch, West Grove, PA) or Alexa488-conjugated secondary antibodies. Omission of the primary antibody eliminated all staining, but nonspecific fluorescence often appeared in the photoreceptor layer.

For Figure 3 the following procedure was used. Eyecups of zebrafish were dissected in L-15 Leibowitz medium, fixed with 2% paraformaldehyde in 0.1 M phosphate buffer (PB), pH 7.4, for 20 min, washed several times in 0.1 M PB, subjected to cryoprotection (30% sucrose in 0.1 M PB) overnight, and embedded in Cryoblock (Medite, Germany) at −20°C. Cryosections (18 µm) of wild-type and C54X mutant zebrafish were placed side by side on gelatine-coated coverslips and further treated under identical experimental conditions. In brief, after three washes (20 min each) in Tris-buffered saline (50 mM Tris, 1.5% NaCl), pH 7.4, containing 0.3% Triton X-100 (TBSTx-buffer) sections were blocked with 10% normal goat serum (NGS, Sigma, Deisenhofen, Germany) in TBSTx-buffer at room temperature for 1 h. Incubations with primary antibodies diluted (anti-Cx55.5: 1∶3,000, anti-Cx52.6: 1∶500, anti-Cx52.9: 1∶200, anti-Fret43: 1∶750) in TBSTx-buffer were carried out at 4°C overnight. Sections were rinsed in TBSTx-buffer, before secondary antibodies (Alexa488-conjugated goat anti-rabbit IgG, Alexa568 goat anti-mouse IgG, Invitrogen, Karlsruhe, Germany; or FITC-conjugated goat anti-chicken IgY, Jackson Immunoresearch, West Grove, PA) diluted 1∶600 in TBSTx-buffer containing 2% NGS, were applied at room temperature for 2 h. Finally, sections were subjected to three washes (20 min each) in TBSTx-buffer and mounted in Vectashield (Vector Laboratories, Burlingame, CA).

### Electronmicroscopy–Tannic Acid Staining

Zebrafish eyes were fixed in a 0.1 M sodiumcacodylate buffer (pH 7.6) solution of 1% tannic acid and 2.5% glutaraldehyde. Eyes were fixed during 1 h. Retinas were isolated, rinsed in sodium cacodylate buffer (0.1 M, pH 7.6), and were fixed for 1 h in 1% osmium solution containing 1.5% potassiumferrocyanide in 0.1 M sodium cacodylate buffer (pH 7.6). Retinas were dehydrated and embedded in epoxy resin. Ultrathin sections were made and examined in a FEI electron microscope.

### Production of Photomicrographs

Light micrographs were acquired as TIFF files directly from the Leica microscope with a Leica 350 F digital camera at 1,284×1,028 pixels. Confocal micrographs were collected and digitized as TIFF files with a Zeiss LSM510 or a Leica TCS SL confocal microscope and software package at a resolution of 1,024×1,024 or 1,024×256 pixels. The same intensity and size thresholds were used for all images taken from the sections of wild-type and mutant zebrafish retinas on the same day. Images were taken from retinal cryosections cut within ∼1 mm of the optic nerve and are presented as projections of stacks of 11×0.2 µm scans (2.2 µm thickness). To analyze the distribution of the various connexins and markers, images were adjusted in brightness and contrast using Photoshop 7.0 (Adobe, San Jose, CA). Electron micrographs were acquired as TIFF files with a MegaView II TEM CCD camera and image acquisition software package (Soft Imaging System, Münster, Germany).

### Quantification of Morphology

Dapi staining was used to quantify the density of the nuclei in the outer nuclear layer (ONL), inner nuclear layer (INL), and the ganglion cell layer (GCL) in wild-type and mutant retinas. Sections from at least five retinas of each group were analyzed. The surface of the layers and nuclei in ONL, INL, and GCL were segregated and counted using Image Pro (Media Cybernetics, Bethesda, MD). Gap-junctions were visualized at the EM level with the gap-junction specific antibody [44]. The length of 50 labeled gap-junctions were measured in both wild-type and mutant retinas using Image Pro (Media Cybernetics, Bethesda, MD). Serial sections (80–100 µm) were made from the wild-type and mutant retinas. Every second section was photographed. Seven series of sections from various wild-type retinas and seven series of sections from various mutant retinas were reconstructed. Only gap-junctions were counted that appeared first in the sections.

### Quantitative PCR (qPCR)

The method and all reagents used have been described in detail previously [49]. Upstream primers and reverse primers were designed using VectorNTI software (Life Technologies Corporation). Amplification reactions were performed in triplicate with each primer pair producing single amplification products of a calculated Tm which was verified by melting point analysis. Syber Green I reaction conditions were as recommended by the manufacturer (Clontech Mountain View, CA, USA) using the DNA Engine Opticon 2 Real-Time PCR Detection System (Bio-Rad Laboratories GmbH, München, Germany). The threshold cycle (C_t_) was defined at the point where the fluorescence signal reached a value of 0.01 above background during the exponential phase of the reaction. The average Ct values were used to calculate the ratios using equation (1). Ratios (R) represented the relative expression of a target gene in a sample:
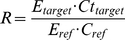
(1)Primer efficiencies (E) of the PCR reactions were determined directly from amplification curves using the DNA Engine Opticon 2 Real-Time PCR Detection System software. The Ct values for the reference gene, 16s rRNA, ß-actin, calbindin, PKCα, vGAT, and GRIAA, were used to normalize mRNA levels of the samples. The changes of the mutant mRNA expression levels were calculated as ratios relative to the mRNA levels found in wild-type fish. All experiments represented three independent sets of samples analyzed in triplicate. Intra-assay and inter-assay variabilities were less than 3%. Statistical analysis was performed using the Relative Expression Software Tool (REST) software [50]. For details of the primers used see Table 1.

**Table 1:** Summary of reference primers used for qRT-PCR of wild-type and mutant fish.

Gene	Primer Sense [5′-3′]	Primer Antisense [5′-3′]	Amplicon Size [bp]	Accession Number
Calbindin	aaacgcgtacctgcagggagtagag	cgcgtacctgcagggagtagag	103	XM_001341568
PKCα	tacagatcaatgactcgtccgccg	tgggctgcttgaagaagcgagc	125	XM_001338950
vGAT	tacctgacgtgggctgatgaaacc	tcttttcgaggacctccaccgc	147	BC129202
GRIAA	ccatgaacgagtatatcgagcagcg	ggcgttcttaatgaggatcctttgg	136	NM_131894
ß-actin	atggatgaggaaatcgctgcc	tgatgtctgggtcgtccaacaatg	121	NM131031
16s rRNA[49]	agataaacctctaccttcggttggg	gttctgaggcttagagatgtttctcttgg	106	AC024175

### Retina Preparation

#### Isolated retina

All steps were carried out in the presence of dim ambient light (room lights off). Zebrafish were dark-adapted in a light-tight chamber for 7 to 10 min, euthanized by immersion in ice water, and decapitated. The head was bisected along the anterior/posterior axis, and the eyes were removed and placed in cold Leibovitz's L-15 medium (Invitrogen, SKU# 11415-064). An eyecup was prepared under red light illumination by removing the cornea, lens, and as much vitreous humor as possible. The eyecup was bisected with a scalpel, and each half-retina was removed from the eyecup with a fine paint brush. Isolated half-retinas were mounted (ganglion cell layer down) to a small piece of kimwipe (Kimberley-Clark Corporation) or lens paper (Pelco lens paper, Catalog # 810, Ted Pella, Inc., Redding, CA) and placed in a recording chamber. The ends of the lens paper/kimwipe were held securely in place under a harp or under two pieces of plastic or Sylgard (Dow Corning Sylgard 184 Silicone Elastomer Kit) that were adhered to the bottom of the chamber with Dow Corning silicone vacuum grease (Electron Microscopy Sciences, Catalog #60705, Hatfield, PA). Retinas mounted in this manner were immersed in cold L-15 medium and kept at approximately 15°C until use in electrophysiological experiments.

#### Dissociated horizontal cells

Adult zebrafish (*Danio rerio*) were kept in a 14∶10 (light/dark) cycle and euthanized in accordance with the National Institutes of Health guidelines for animal use. Dark adapted retinas were removed and then incubated in Leibovitz's L-15 medium (Invitrogen) containing 20 U/ml papain (Worthington) activated with 0.3 mg/ml cysteine. Penicillin/Streptomycin (1∶100, Sigma) was routinely added to prevent bacterial growth. Retinas were incubated in L-15/papain solution for 10 min followed by six changes of fresh L-15 medium, and then dissociated by repeated passage through a trimmed 1-ml pipette tip. Cultures were maintained at 20°C and cells were recorded within 1–2 d of dissociation.

### Electrophysiology—Voltage Clamp Measurements of Cones

#### Electrodes and recording equipment

Whole-cell voltage clamp recordings were obtained from cones. Recording chambers containing isolated retina preparations were mounted on a Nikon Optiphot-X2 microscope (Nikon, Japan) or a Zeiss Axio Scope (Carl Zeiss Microimaging, Inc., Thornwood, NY). Retinal preparations on the Nikon microscope were magnified with a Nikon 60× water immersion objective with differential interference contrast and viewed with infrared light with a video camera (Philips, The Netherlands). Recording electrodes were pulled from borosilicate glass (GC150TF-10; Harvard Apparatus, Holliston, MA) with a Flaming/Brown micropipette puller (Model P-87; Sutter Instruments Company) or a Narishige micropipette puller (Narishige, East Meadow, NY). The impedances ranged from 3 to 6 MΩ when filled with pipette medium and measured in bathing solution. Signal software (v. 3.07; Cambridge Electronic Design (CED), Cambridge, UK) or pClamp 6.0 (Molecular Devices, Sunnyvale, CA) was used to generate voltage command outputs and to acquire data. The data were digitized and stored with a PC using a CED Micro1401 mkII AD/DA converter with expansion ADC12 module (used with Signal Software) or with the Digidata 1300 (Molecular Devices, Sunnyvale, CA; used with pClamp 6.0 Software). Responses were filtered at 1 kHz on a DAGAN 3900A integrating patch clamp (Dagan, Minneapolis, MN; used with MS-DOS and Signal Software) or on an Axopatch 200B (Molecular Devices, Sunnyvale, CA; used with pClamp 6.0 Software). Signal software (v. 3.07; CED), Sigma Plot software (demo version 9.0; SPSS, Chicago, IL), and Origin Pro 7.5 (Origin Lab Corporation, Northampton, MA) were used to analyze the electrophysiological recordings and perform calculations.

#### Electrode and bath solutions

The standard patch pipette medium contained (in mM): 9 KCl, 86.4 K-gluconate, 1 MgCl_2_, 0.1 CaCl_2_, 2 EGTA, 5 4-(2-Hydroxyethyl)piperazine-1-ethanesulfonic acid, N-(2-Hydroxyethyl)piperazine-N′-(2-ethanesulfonic acid) (HEPES), 5 Adenosine 5′-triphosphate dipotassium salt dihydrate (ATP-K_2_), 1 Guanosine 5′-triphosphate sodium salt hydrate (GTP-Na*), 0.2 Guanosine 3′,5′-cyclic monophosphate sodium salt (cGMP-Na), 17 phosphocreatine-Na_2_, and 50 units/ml creatine phosphokinase. The pH of the pipette medium was adjusted to 7.3 to 7.4 with KOH. The bath solution contained in (mM): 102 NaCl, 3 KCl, 2 CaCl_2_, 1 MgCl_2_, 5 D-glucose, and 28 NaHCO_3_. All chemicals were obtained from Sigma-Aldrich (St Louis, MO). In experiments with the standard patch pipette medium, E_Cl_ was calculated to be approximately −59 mV. The liquid junction potential was calculated to be approximately 17.5 mV. The bath solution was continuously gassed with a mixture of CO_2_/O_2_, yielding a pH of 7.4 to 7.5. Bath solution flowed continuously through the recording chamber via a gravity-driven superfusion system. Switching between bathing solutions was achieved through computer-controlled or manual opening and closing of valves. During all electrophysiological experiments with the isolated retina, GABAergic transmission in the retina was blocked by 200 µM PTX. PTX, DNQX, and KA were obtained from Sigma-Aldrich.

#### Measuring light-induced feedback in cones

Whole cell voltage clamp recordings were made from cones in flat-mount dark-adapted retinas. Cones were clamped at their resting membrane potential (−45 mV to −55 mV). The cone type was determined with 500 ms stimuli of different wavelengths (465 nm, 525 nm, and 624 nm). The cone was stimulated with a saturating spot of light (20 µm diameter) of the wavelength that yielded the largest response. Subsequently, a full-field stimulus of white light (1.0 or 1.5 s) was given of increasing intensities, while the cell was stepped to −30 mV. Amplitudes of feedback responses were measured at 200 ms after response onset. Cells, in which the Calcium-dependent Chloride current confounded the measurements, were discarded.

#### Measuring maximal feedback in cones

Feedback from horizontal cells to cones shifts the Ca^2+^-current (I_Ca_) of the cone to more negative potentials [1]. In a voltage-clamped cone, this shift can be seen as an increase in *I_Ca_* at physiological potentials, which leads to an increase in glutamate released by the cone. The I–V relation of the *I_Ca_* was obtained by leak subtracting the cone I–V relation; the leakage current was estimated from the linear portion of the I–V curve, between −100 and −80 mV. To quantify feedback in cones, the inducible shift of *I_Ca_* was determined by hyperpolarizing horizontal cells maximally with DNQX or by depolarizing them with KA. To determine the magnitude of the shift of I_Ca_, the control I–V relation was subtracted from I–V relation in the presence of DNQX or KA and the surface under the resulting curve was calculated. The adaptation condition of the retina and background light condition during these experiments were equal to those for the experiments involving light responses.

#### Voltage clamp recordings of dissociated horizontal cells

Recordings from solitary H1 horizontal cells were performed using the conventional whole-cell patch-clamp configuration. The normal Ca^2+^ bath solution contained (in mM): 137 NaCl, 2.5 KCl, 2.5 MgCl_2_, 2.5 CaCl_2_, 10 HEPES, 10 glucose, and 1 mg/ml BSA (Sigma, Fraction VII), and pH was adjusted to 7.5 using NaOH. The Ca^2+^-free bath solution contained (in mM) 114.5 NaCl, 2.5 KCl, 1 MgCl_2_, 10 HEPES, 30 CsCl, 1 Na-pyruvate, 10 glucose, and 1 mg/ml BSA, and pH was adjusted to 7.5 with NaOH. To block potassium channels, K^+^ in the normal pipette solution was replaced by Cs^+^, and tetraethylammonium chloride (TEA) was added to the pipette solution. The pipette solution contained (in mM): 124 CsCl, 1 CaCl_2_, 11 EGTA, 10 HEPES, 1 Mg-ATP, 0.1 Na-GTP, and 10 TEA, pH to 7.5 with CsOH. Patch pipettes were pulled from Corning 7052 glass (AM Systems) and fire-polished to a resistance of 6–10 MΩ. The pipette series resistance and capacitance were compensated by 80%. The offset potential between the pipette and the bath solution was zeroed prior to seal formation. Voltage commands and data analysis were performed using pCLAMP 9.0 software. The amplitude of macroscopic hemichannel currents was read as steady-state whole cell current in the last 10 ms of the voltage steps in low Ca^2+^ medium and with K^+^ channel blockers, as described above. The inward hemichannel current remains 80%–50% active in the physiological range of Ca^2+^ concentrations (1–2 mM) (Sun et al., submitted).

### Current Clamp Recordings of Horizontal Cells

#### Optical stimulator

Optical stimuli of 3 wavelengths (624, 525, and 465 nm) were generated by LED light sources and filtered by neutral density filters (Nikon, Japan). The full-field chromatic light stimuli were projected onto the retina from below through the condenser of the microscope. Spots of light of various sizes were projected onto the retina through the objective of the microscope. The light intensity of the stimulation was 8.5×10^15^ quanta sec^−1^ m^−2^ for all wavelengths.

#### Electrodes and recording equipment

Microelectrodes were pulled on a horizontal puller (Sutter P-80-PC; San Rafael, USA) using aluminosilicate glass (OD = 1.0 mm, ID = 0.5 mm; Clark, UK) and had impedances ranging from 300–400 MΩ when filled with 3 M KCl. The intracellular recordings were made with a WPI S7000A microelectrode amplifier system (World Precision Instruments, USA) and sampled using an AD/DA converter (CED 1401, Cambridge Electronic Design, UK) coupled to a Windows-based computer system.

The roll-back response or secondary depolarization in the light response of horizontal cells has been used as an indirect measure of feedback from horizontal cells to cones [18]–[21]. The size of the roll-back response was determined as the difference between the initial response 100–150 ms after onset of the stimulus and its amplitude 500 ms after the onset of the stimulus. The amount of roll-back is expressed as the ratio between the roll-back response and the response amplitude. In biphasic horizontal cells, feedback is observed as a depolarizing response to red light stimulation [19],[21],[24],[51],[52]. The depolarizing response to red light in biphasic horizontal cells is considered as a direct measure of feedback strength.

### Model Description

The model describing negative feedback from horizontal cells to cones (Figures 7 and 8) is an extended version of a model described for goldfish retina [6]. Briefly, a simple conductive network (Figure 7) was used to evaluate whether the physiology and morphology of the cone/horizontal cell synapse allows for physiologically relevant ephaptic interaction. The horizontal cell is modeled as three conductances with their associated reversal potentials; the hemichannel conductance (*g_hemi_*), the glutamate gated conductance (*g_Glu_*) and a non-linear potassium conductance (*g_k_*) taken from [53]. *g_hemi_* is located on the dendrites of horizontal cells, while *g_Glu_* is located on both the dendrites and the soma of the horizontal cells. The reversal potentials for *g_hemi_* and *g_Glu_* are 0 mV and that for *g_K_* is −82.7 mV (*E_K_*). In the dark, the horizontal cell membrane potential (*V_HC_*) is more positive than *E_K_*, and current will flow from *g_K_* into *g_hemi_* and *g_Glu_* via an extracellular resistive pathway in the synaptic complex whose conductance is (*g_ext_*). This current will generate a voltage drop over *g_ext_*, making the potential deep in the synaptic cleft (*V_ext_*) slightly negative. The light-induced closure of *g_Glu_* causes the horizontal cell to hyperpolarize, resulting in a change in the current through *g_ext_*, and a greater negativity in *V_ext_*. The fall in *V_ext_* serves to depolarize the cone membrane locally, modulate the cone Ca^2+^-current (*I_Ca_*), and increase the release of neurotransmitter. All parameters and equations of the model were kept equal to parameter set 2 in Fahrenfort et al. [6]. In order to compare the resulting changes of voltage in the synaptic cleft, the model was extended with a reduced cone model. The cone model consisted only of a voltage dependent Ca-current and glutamate release. *I_Ca_* was modeled according to equation (2) [54] with parameters that fitted best our measurements of *I_Ca_* in DNQX in Figure 6B, after shifting it over the value of *V_ext_* that is given by horizontal cell model for *g_Glu_* = 0 nS. This fit yields the following parameter values: *K_Ca_* = −5.4 mV, *E_Ca_* = 44.6 mV, *n_Ca_* = −12 mV and 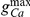 = 36 (normalized units). The glutamate conductance in the horizontal cell (*g_Glu_*) was adapted from a fixed parameter to a variable. The relation between the calcium current *I_Ca_* in the cone and the glutamate dependent conductance *g_Glu_* in the horizontal cell is described by equation (3). This relation is based upon the finding that the glutamate release depends linearly on *I_Ca_* [55] and that the dependence of *g_Glu_* on the glutamate concentration can be described by a Hill function with coefficient 2 [55],[56]. We have used 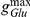 = 10 nS and *K_Glu_* = 0.1 (normalized units), as for those values the simulations of the feedback model show the best correspondence with the wild-type measurements shown in Figure 6B.
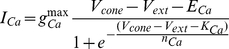
(2)

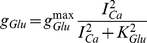
(3)The present extension of the model consists of only one free parameter: *K_Glu_*. To determine how critical this value is for the model behavior, we varied *K_Glu_* over a wide range and found that the model showed the same overall behavior for all values tested. The DNQX-induced feedback currents always reduced with reduction of *g_hemi_* and finally reversed whereas the KA induced feedback current only reduced slightly with the reduction of *g_hemi_* . Some differences between the model and the actual data exist especially at potentials more positive than 0 mV. These differences might be due to the absence of voltage or Ca^2+^-gated currents in the cone model. Overall the model fully captures the highly specific modification of feedback from horizontal cells to cones due to the reduction of *I_hemi_*. Since this behavior is highly independent of the parameter values, it can be considered as a hall-mark of hemichannel mediated feedback.

### Optokinetic Response

The optokinetic response (OKR) was measured at an age of 5 to 6 d post-fertilization (dpf). The OKR is a reflex which is triggered by objects moving across the visual field. The OKR consists out of slow smooth pursuit eye movements interrupted by fast saccadic movements that reset the eyes. The optokinetic stimulator is formed by 60 circularly placed plexiglass slides. Each slide is illuminated by 5 LEDs at the top and 5 LEDs at the bottom (one of 630 nm, two of 526 nm, and the two of 458 nm). The intensity of the LEDs can be updated every 4 ms. In that way moving stimuli were generated (Figure S3). A zebrafish larva was put, dorsal side upwards, into a Petri dish with a diameter of 1.35 cm in the center of the optokinetic drum. By immerging the larvae in 3% methylcellulose, all body movements except the eye movements were suppressed. The fish were illuminated with IR-light from below and eye movements were determined using labview software (kind gift of S. Neuhauss). The sine-wave stimulus used in the experiments shown in Figure 9A and B had a spatial frequency of 0.028 cycles per degree, contrasts of 100%, 50%, 25%, 12.5%, and 6.25%, mean intensity 16 lux, and a temporal frequency of 0.25 cycles per second. The sine-wave stimulus used in the experiments of Figure 9C had a spatial frequency of 0.014 cycles per degree, a contrast of 100%, and a temporal frequency of 0.25 and 1.0 cycles per second. To avoid habituation of the response, the stimulus first moves counterclockwise for 12 s, then holds still for 3 s before it moves clockwise for 12 s (Figure 9A). Contrast is defined by equation (4).
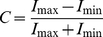
(4)The optokinetic gain is defined as the mean velocity of the slow pursuit movements of both eyes in both directions divided by velocity of the stimulus. A gain of 1.0 would mean that the image is stabilized onto the retina. The surface under the contrast/gain curves was determined for each individual fish. The means of these surfaces were compared for wild-type and mutant.

### Statistical Analysis

Data are presented ± the standard error of the mean (SEM). Levels of significance were determined using Student's *t* test or a Pearson's χ^2^ test. Data were considered significantly different for *p* values smaller than 0.05. Curve fitting and statistical analyses were performed using SPSS, Sigmaplot 10.0 or Origin 8.0 software.

## Supporting Information

Figure S1 Effects of Co^2+^ on macroscopic hemichannel currents of isolated zebrafish horizontal cells. (A) Representative current trace elicited in Ca^2+^-free medium before (left) and after application of 100 µM Co^2+^ (right). Time and amplitude are indicated on the scale bar. (B) Normalized currents obtained in Ca^2+^-free medium (open bar) and in medium containing 100 µM Co^2+^ (closed bar). Outward and inward currents were normalized to the currents elicited in Ca^2+^-free medium at +60 mV and −60 mV, respectively (*n* = 5, mean ± SEM). 100 µM Co^2+^ significantly blocked the outward currents to 46±5% of control (*p*<0.001, *n* = 5) and decreased the inward currents to 53±6% of control (*p*<0.001, *n* = 5). *** *p*<0.001.
(TIF)

Figure S2 Amplitudes of depolarizing responses in biphasic horizontal cells are strongly reduced in mutant zebrafish. Biphasic horizontal cells depolarize to red light stimuli and hyperpolarize to blue light stimuli. In zebrafish one can find biphasic horizontal cells that either hyperpolarize (left) or depolarize (right) in response to green light stimuli. In both types of BHCs, all depolarizing responses are smaller in the mutant.
(TIF)

Figure S3 LED based optokinetic stimulator. (A) LED based optokinetic stimulator generating sine-wave patterns. Zebrafish larvae are positioned in the middle of the stimulator and are illuminated from below with infrared light. A video camera is positioned directly above the larvae and focused on the eyes of the fish. (B and C) Plexiglass slides, which can be illuminated by red, green, and blue LEDs, are assembled on circuit-boards. These circuit boards are arranged in a circle. For demonstration purposes, only 4 of the 60 plexiglass slides are installed in these images. (D) Video image of a zebrafish larva in the optokinetic stimulator.
(TIF)

## Abbreviations

BC: bipolar cell
BHC: biphasic horizontal cell
DNQX: 6,7-Dinitroquinoxaline-2,3(1H,4H)-dione
GCL: ganglion cell layer
KA: Kainate
INL: inner nuclear layer
IPL: inner plexiform layer
MHC: monophasic horizontal cell
OKR: optokinetic response
ONL: outer nuclear layer
OPL: outer plexiform layer
THC: triphasic horizontal cell
WT: wild-type
