# Supplementary figures and images for: Synaptic Transmission from Horizontal Cells to Cones Is Impaired by Loss of Connexin Hemichannels
Source: PLoS Biol. 2011 Jul 19;9(7):e1001107. doi: 10.1371/journal.pbio.1001107 (PMC3139627; doi:10.1371/journal.pbio.1001107)

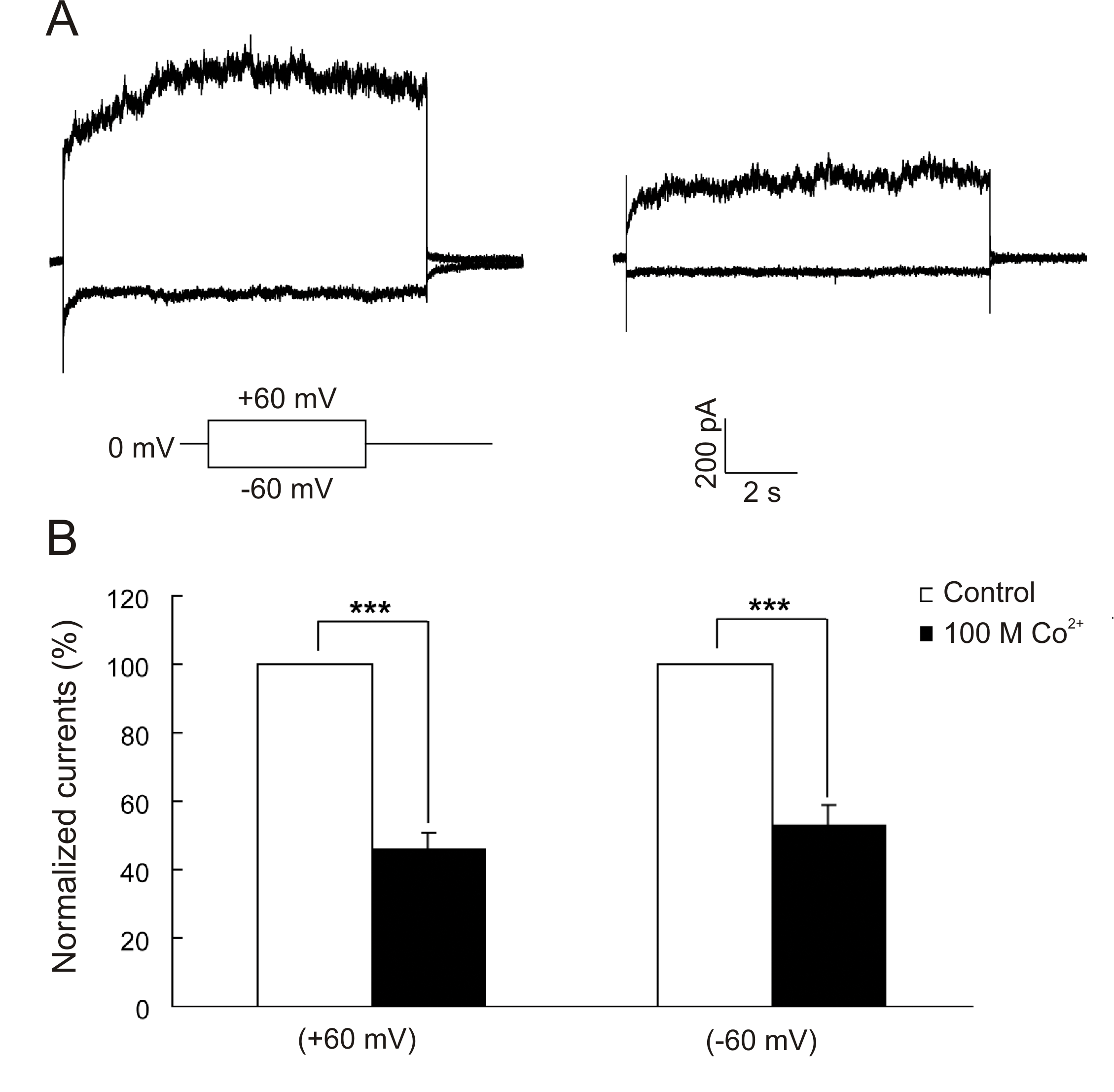

Supplement: Figure S1 — Effects of Co2+ on macroscopic hemichannel currents of isolated zebrafish horizontal cells. (A) Representative current trace elicited in Ca2+-free medium before (left) and after application of 100 µM Co2+ (right). Time and amplitude are indicated on the scale bar. (B) Normalized currents obtained in Ca2+-free medium (open bar) and in medium containing 100 µM Co2+ (closed bar). Outward and inward currents were normalized to the currents elicited in Ca2+-free medium at +60 mV and −60 mV, respectively (n = 5, mean ± SEM). 100 µM Co2+ significantly blocked the outward currents to 46±5% of control (p<0.001, n = 5) and decreased the inward currents to 53±6% of control (p<0.001, n = 5). *** p<0.001. (TIF) [file pbio.1001107.s001.tif]

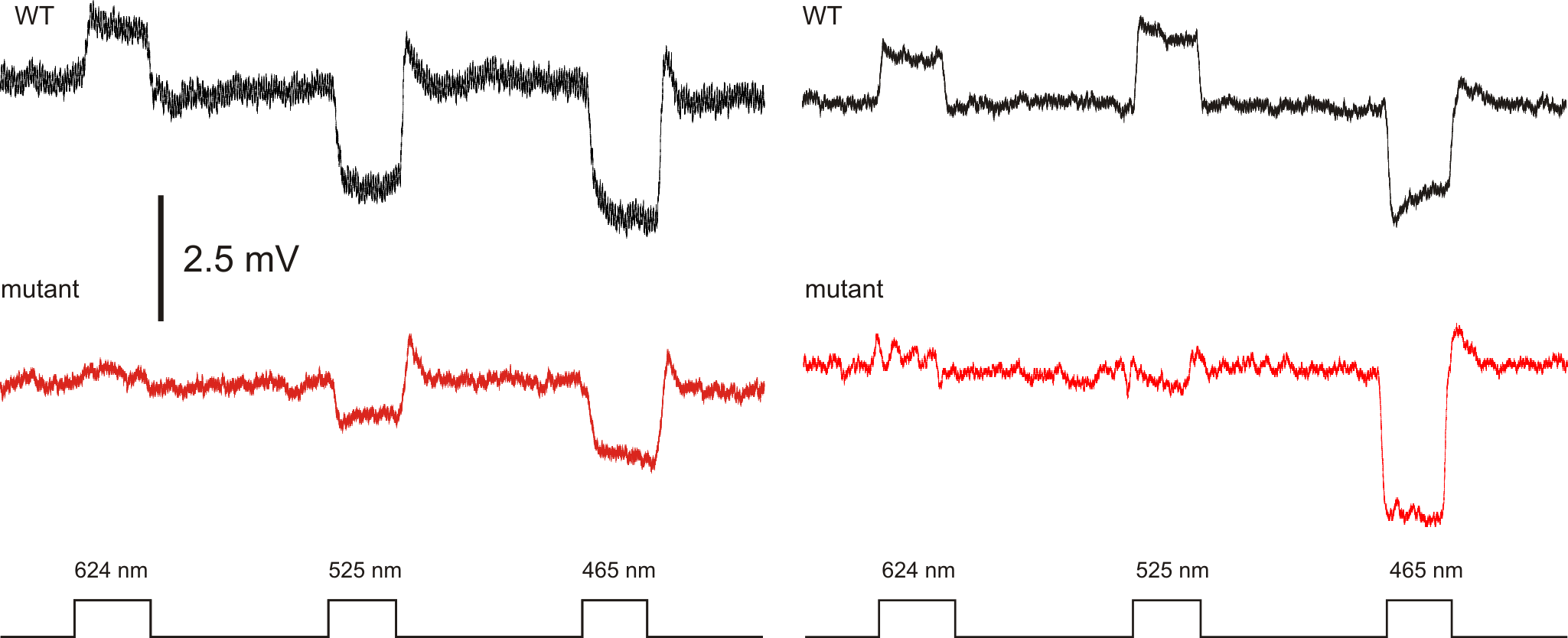

Supplement: Figure S2 — Amplitudes of depolarizing responses in biphasic horizontal cells are strongly reduced in mutant zebrafish. Biphasic horizontal cells depolarize to red light stimuli and hyperpolarize to blue light stimuli. In zebrafish one can find biphasic horizontal cells that either hyperpolarize (left) or depolarize (right) in response to green light stimuli. In both types of BHCs, all depolarizing responses are smaller in the mutant. (TIF) [file pbio.1001107.s002.tif]

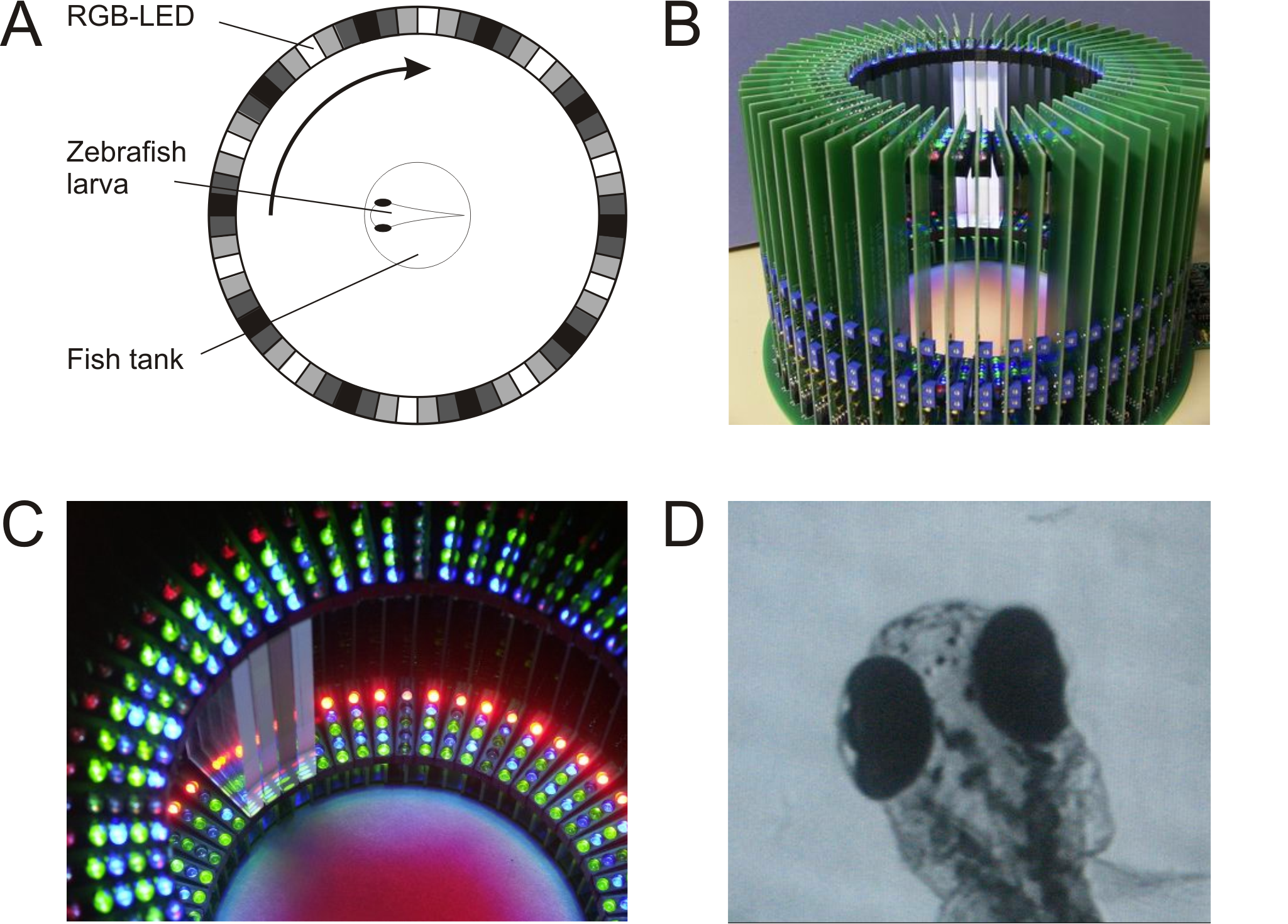

Supplement: Figure S3 — LED based optokinetic stimulator. (A) LED based optokinetic stimulator generating sine-wave patterns. Zebrafish larvae are positioned in the middle of the stimulator and are illuminated from below with infrared light. A video camera is positioned directly above the larvae and focused on the eyes of the fish. (B and C) Plexiglass slides, which can be illuminated by red, green, and blue LEDs, are assembled on circuit-boards. These circuit boards are arranged in a circle. For demonstration purposes, only 4 of the 60 plexiglass slides are installed in these images. (D) Video image of a zebrafish larva in the optokinetic stimulator. (TIF) [file pbio.1001107.s003.tif]
